# Fatty acyl-CoA reductases of birds

**DOI:** 10.1186/1471-2091-12-64

**Published:** 2011-12-12

**Authors:** Janine Hellenbrand, Eva-Maria Biester, Jens Gruber, Mats Hamberg, Margrit Frentzen

**Affiliations:** 1 Special Botany, Institute for Biology I, RWTH Aachen University, Aachen, Germany; 2 Department of Medical Biochemistry and Biophysics, Division of Physiological Chemistry II, Karolinska Institute, Stockholm, Sweden

## Abstract

**Background:**

Birds clean and lubricate their feathers with waxes that are produced in the uropygial gland, a holocrine gland located on their back above the tail. The type and the composition of the secreted wax esters are dependent on the bird species, for instance the wax ester secretion of goose contains branched-chain fatty acids and unbranched fatty alcohols, whereas that of barn owl contains fatty acids and alcohols both of which are branched. Alcohol-forming fatty acyl-CoA reductases (FAR) catalyze the reduction of activated acyl groups to fatty alcohols that can be esterified with acyl-CoA thioesters forming wax esters.

**Results:**

cDNA sequences encoding fatty acyl-CoA reductases were cloned from the uropygial glands of barn owl (*Tyto alba*), domestic chicken (*Gallus gallus domesticus*) and domestic goose (*Anser anser domesticus*). Heterologous expression in *Saccharomyces cerevisiae *showed that they encode membrane associated enzymes which catalyze a NADPH dependent reduction of acyl-CoA thioesters to fatty alcohols. By feeding studies of transgenic yeast cultures and *in vitro *enzyme assays with membrane fractions of transgenic yeast cells two groups of isozymes with different properties were identified, termed FAR1 and FAR2. The FAR1 group mainly synthesized 1-hexadecanol and accepted substrates in the range between 14 and 18 carbon atoms, whereas the FAR2 group preferred stearoyl-CoA and accepted substrates between 16 and 20 carbon atoms. Expression studies with tissues of domestic chicken indicated that FAR transcripts were not restricted to the uropygial gland.

**Conclusion:**

The data of our study suggest that the identified and characterized avian FAR isozymes, FAR1 and FAR2, can be involved in wax ester biosynthesis and in other pathways like ether lipid synthesis.

## Background

Fatty acyl-CoA reductases (FAR) can be divided into two classes that differ with respect to the end-product synthesized, i.e. the aldehyde- and the alcohol-forming enzymes [1]. Aldehyde-generating FAR catalyze a two-electron reduction of activated fatty acids, so that fatty aldehydes are formed. Such enzymes have been described in pea leaves, green algae and bacteria [2-6]. The fatty aldehydes can be further reduced to fatty alcohols by fatty aldehyde reductases [7] or can be involved in the biosynthesis of hydrocarbons [5,6]. On the other hand, alcohol-forming FAR catalyze the reduction of activated fatty acids to fatty alcohols. This four-electron reduction takes place in two steps. In the first step an aldehyde is formed, that is subsequently reduced to a fatty alcohol in the second step [1]. Proteins have been purified and genes encoding alcohol-forming FAR were identified in plants [1,8-11], mammals [12], insects [13-17], birds [18] and protozoa [19]. They usually require NADPH as electron donor but in certain organisms like *Euglena gracilis *[20] NADPH can be substituted by NADH.

FAR enzymes of plants are involved in the biosynthesis of cutin, suberin and storage lipids [1,8,10]. In some insect species long-chain alcohols function as sex or communication pheromones [14,21]. Mammalian FAR enzymes produce alcohols needed for the biosynthesis of wax esters and ether lipids [12,22-24] but in the preputial gland long-chain alcohols like 1-hexadecanol can serve as putative chemical signals in sex recognition [25,26]. In birds fatty alcohols are constituent parts of wax esters that are used for cleansing and lubricating of their feathers. The waxes are accumulated in a special holocrine gland, the uropygial gland, that lies between the fourth caudal vertebra and the pygostyle [27]. Type and composition of these wax esters are dependent on the bird species. For instance, the wax ester secretion of goose contains multi-methyl-branched fatty acids and straight-chain fatty alcohols [28,29], whereas that of barn owl can contain both, methyl-branched fatty acids and alcohols [30]. The secretion of the uropygial gland from chicken is composed of 2,3-diester waxes, whose biosynthesis is not completely elucidated yet [31-33]. Furthermore it was demonstrated that fatty alcohols are possibly involved in the chemical communication of budgerigars [34] and that uropygial glands can play a role in the sexual behavior in domestic chicken [35].

Fatty alcohols in the range between 6 to 22 carbon atoms are used to produce lubricants, emulsifiers, agrochemical formulations, pharmaceutical and cosmetic products [36]. For instance, in Western Europe about 454 thousand tonnes of fatty alcohols were used in 2006 to produce alcohol ethoxylates [37]. Production of these alcohols from petrochemical raw material will consume remaining fossil oil resources, hence there is the need of inventing new production technologies in future. For instance, metabolic engineering of oil crops was carried out to increase the yield of fatty acids and to modify the fatty acid composition, so that these crops could achieve the requirements of the producing chemical industry and replace fossil oil [38].

In order to identify new fatty alcohol synthesizing enzymes with catalytic activities suitable for the producing chemical industry, we investigated the uropygial glands of barn owl, domestic chicken and domestic goose. In this study we report on the identification of avian FAR sequences by similarity-based sequence search, on the heterologous expression of the genes in yeast, and on the catalytic properties of the recombinant enzymes.

## Results

### Identification and isolation of FAR sequences from uropygial glands

Based on sequence similarity with FAR proteins of mammals and jojoba, two putative FAR sequences of chicken, FAR1 and FAR2 (additional file 1), were identified. To clone the cDNA sequences, mRNA was isolated from the uropygial glands and used to amplify the FAR sequences by RT-PCR with gene specific primers. Resulting cDNAs were analyzed and finally cloned into yeast expression vectors. Analyses of the cDNA sequences from chicken showed that they encoded amino acid sequences with conservative substitutions in one (FAR1: position 254: phenylalanine substituted by tyrosine, termed GgFAR1db) or in two sites (FAR2: position 159: valine substituted by methionine, position 457: lysine substituted by arginine, termed GgFAR2) compared to the database sequences. Furthermore a splicing variant of FAR1 (substitution of position 319 to 376, annotated as exon 9, by intron sequence of the same length; see additional file 2), termed GgFAR1, was identified. Moreover barn owl and goose were found to possess putative FAR sequences very similar to those of chicken. Two cDNA sequences of barn owl, named TaFAR1 and TaFAR2, and one of goose, termed AdFAR1, were additionally amplified from the uropygial glands (additional file 1). In contrast to barn owl and chicken, we failed to clone a FAR2 sequence from the uropygial gland of goose.

Sequence analysis showed that all avian FAR proteins had two conserved domains, the NADB-Rossmann superfamily domain [cd05236: FAR-N_SDR_e] and the FAR_C superfamily domain [cd09071: FAR_C] and one predicted transmembrane region at the C-terminus (additional file 3). FAR enzymes of mammals have been shown to possess domain patterns similar to those of avian FAR while those of plants appear to lack the transmembrane region (additional file 3) [10,12].

**Figure 1:**
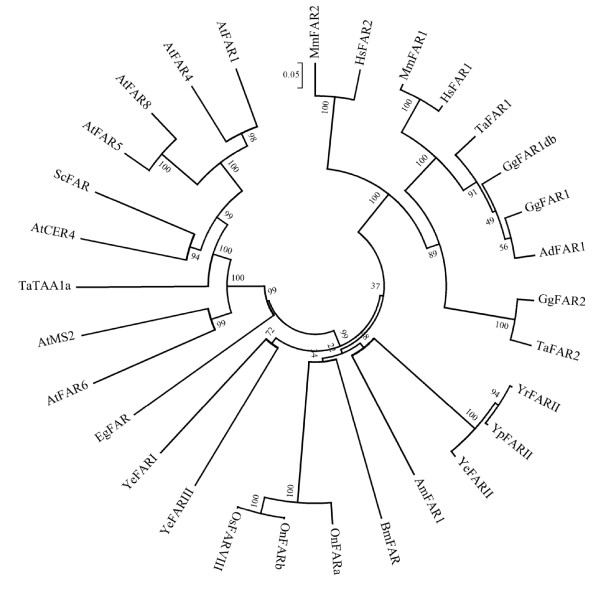
**Evolutionary relationships of FAR from different taxa**. Amino acid sequences of FAR from birds (*Anser anser domesticus*, Ad; *Gallus gallus domesticus*, Gg; *Tyto alba*, Ta), mammals (*Homo sapiens*, Hs; *Mus musculus*, Mm), insects (*Apis mellifera*, Am; *Yponomeuta evonymellus*, Ye; *Yponomeuta rorellus, Yr; Yponomeuta padellus*, Yp; *Ostrinia nubilalis*, On; *Ostrinia scapulalis*, Os; *Bombyx mori*, Bm), protozoa (*Euglena gracilis*, Eg) and plants (*Arabidopsis thaliana*, At; *Triticum aestivum *L., Ta; *Simmondsia chinensis*, Sc) were compared to calculate a bootstrap consensus tree. Numbers above branches: bootstrap values, scale: number of amino acid differences per site.

Phylogenetic analysis of FAR amino acid sequences of birds and other organisms resulted in a bootstrap consensus tree shown in Figure 1. It turned out that the avian sequences could be divided into two groups. AdFAR1, GgFAR1, TaFAR1 and GgFAR1db shared high sequence identity with each other as well as with FAR1 sequences of mammals (89% to 93%). The sequence identity between GgFAR2 and TaFAR2 was in the same range as that between the avian FAR1 proteins (93%). On the other hand FAR2 of birds showed relatively low sequence identities with the avian FAR1 group and the mammalian FAR1 and FAR2 proteins (about 68% at most). The avian sequences showed the lowest identity with FAR proteins of plants with at most 21%.

### Functional expression studies in yeast

**Figure 2:**
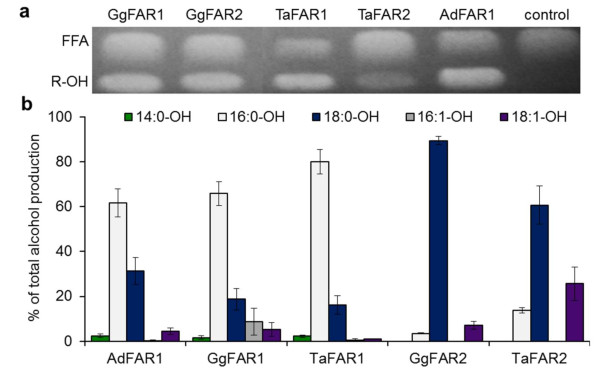
**Alcohol production of yeast strains expressing avian FAR enzymes from endogenous fatty acids**. **(a) TLC analysis of lipid extracts from transgenic yeast cells**. Total lipid fraction of yeast cells expressing one of the avian FAR sequences or the empty vector control were extracted after 72 h incubation and lipid extracts were analyzed by TLC. FFA: free fatty acids, R-OH: fatty alcohols. **(b) Alcohol composition of transgenic yeast strains**. The alcohol composition was analyzed and quantified by GC. Results are mean values of duplicate extractions from two independent yeast cultures. Following concentrations in μmol*g^-1 ^fresh weight correspond to 100%: AdFAR1:15.6, GgFAR1:13.2, TaFAR1: 17.7, GgFAR2: 8.2, TaFAR2: 2.9, control: 0.02. Because GgFAR1db showed results very similar to those of GgFAR1, results of GgFAR1 are presented only.

To verify the catalytic properties of the avian sequences, functional expression studies in yeast were carried out. The data provided clear evidence that these sequences encoded fatty acyl-CoA reductases that catalyzed the production of fatty alcohols (Figure 2a and 2b). TaFAR1 showed the highest activity in yeast and produced up to 18 μmol fatty alcohol per gram fresh weight. The data in Figure 2b also suggest, that the chain length specificity increased from AdFAR1, via GgFAR1 to TaFAR1, while GgFAR2 had a more pronounced substrate specificity than TaFAR2.

**Figure 3:**
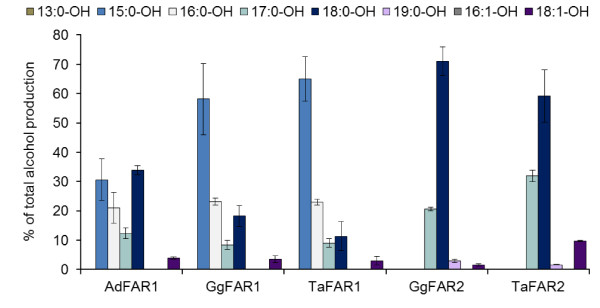
**Alcohol production of yeast strains expressing avian FAR enzymes supplemented with a mixture of odd-numbered saturated fatty acids**. Yeast cells expressing one of the avian FAR proteins or the empty vector control were supplemented with a mixture of 13:0, 15:0, 17:0, 19:0, extracted after 72 h incubation and synthesized fatty alcohols were analyzed by GC. Results are mean values of extractions from at least three independent yeast cultures. Following alcohol concentrations in μmol*g^-1 ^fresh weight correspond to 100%: AdFAR1: 16.1, GgFAR1: 10.6, TaFAR1: 15.8, GgFAR2: 15.6, TaFAR2: 3.9, control: 0.0.

Feeding experiments with mixtures of fatty acids added to the media were carried out to investigate the alcohol production of intact transgenic yeast cells. Supplementing the transgenic yeast strains with even-numbered, saturated fatty acids between 14 to 22 carbon atoms gave results very similar to those shown in Figure 2b. Only FAR1 cells contained slightly higher amounts of 14:0-OH, while FAR2 cells contained distinctly lower amounts of 18:1-OH and trace amounts of 20:0-OH. Feeding experiments with monounsaturated fatty acids of chain lengths between 14 to 22 carbon atoms resulted in no additionally formed fatty alcohol species unlike those with odd-numbered, saturated fatty acids between 13 to 19 carbon atoms. As given in Figure 3, feeding experiments with odd-numbered fatty acids gave a fatty alcohol production within the yeast cells, distinctly different to the respective yeast cells without supplemented fatty acids (Figure 2b). Yeast cells expressing FAR1 enzymes of barn owl, chicken and goose produced 15:0-OH as main product that comprised up to 65% of the total mixture. Furthermore 17:0-OH was detected in all of these yeast cells, although in lower amounts than 15:0-OH and 16:0-OH (Figure 3). On the other hand, yeast strains expressing FAR2 sequences produced mostly 18:0-OH and 17:0-OH while 19:0-OH was formed in low amounts only.

### Investigation of enzymatic properties

**Figure 4:**
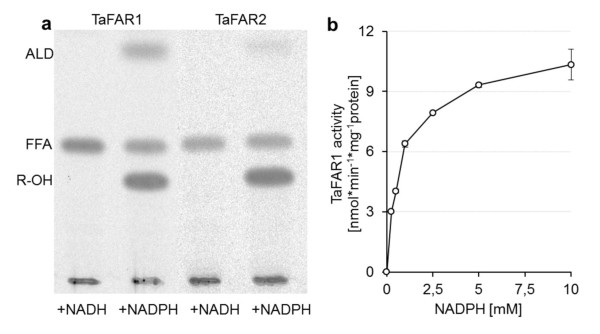
**Cofactor requirement of FAR enzymes.  (a) Cofactor requirement of TaFAR1 and TaFAR2**. Assays were carried out with the total membrane fraction of yeast cells expressing TaFAR1 or TaFAR2. [1-^14^C]-labeled products derived from assays carried out with labeled 16:0-CoA (lane 1 and 2, TaFAR1) or labeled 18:0-CoA (lane 3 and 4, TaFAR2) and NADH (lane 1 and 3) or NADPH (lane 2 and 4). (ALD: fatty aldehyde, FFA: free fatty acid, R-OH: fatty alcohol). **(b) NADPH dependency of TaFAR1**. Assays were conducted with the total membrane fraction of yeast cells expressing TaFAR1 and with [1-^14^C]-labeled 16:0-CoA. The concentration of NADPH was varied under otherwise standard assay conditions. Results are the sum of synthesized aldehydes and alcohols and display mean values of two independent assays.

In line with the predicted transmembrane domain in the C-terminal region of the avian FAR proteins, assays with subcellular fractions of transgenic yeast cells expressing an avian FAR sequence showed that the FAR activity was detectable in the total membrane fraction only. All FAR enzymes required NADPH as electron donor (optimal concentration of about 5 mM, Figure 4) as well as activated acyl groups as substrates (as addition of ATP and CoA to free fatty acids restored FAR activity). Furthermore it was shown that both isoforms produced not only fatty alcohols but also fatty aldehydes as intermediates although in different proportions (Figure 4a, additional file 4). FAR1 enzymes produced distinctly higher aldehyde levels than FAR2 enzymes. In addition, aldehyde production varied with the chain length of the acyl-CoA and was highest in FAR1 assays with 14:0-CoA (additional file 4). These data suggest that the aldehydes are prematurely released intermediates of the FAR reaction. The enzymatic activities were constant for up to 15 min at 37°C. The pH-optimum of FAR1 was found to be at 6.5 while FAR2 displayed the highest activity at about pH 5.5 (additional file 5a). Addition of up to 1 mM MgCl_2 _stimulated the FAR activity 2-fold, while addition of BSA into assays with 20 μM acyl-CoA gave severalfold higher FAR activities (additional file 5b). The alcohol formation rate was constant up to 2 μg protein of membrane fraction with FAR1 and up to 20 μg protein with FAR2 (additional file 5c). Under optimal conditions membranes harboring TaFAR1 showed a specific activity of about 9 nmol product*min^-1^*mg^-1 ^protein whereas TaFAR2 showed a lower activity of about 3 nmol product*min^-1^*mg^-1 ^protein.

**Figure 5:**
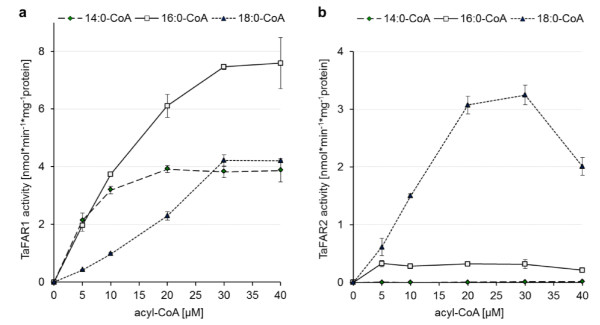
**Acyl-CoA dependencies of TaFAR1 (a) and TaFAR2 (b)**. The total membrane fractions of yeast cells expressing TaFAR1 or TaFAR2 were used as enzyme source. Different concentrations of the given [1-^14^C]-labeled acyl-CoA species were tested under optimal assay conditions. Results are the sum of synthesized aldehydes and alcohols and display mean values of two independent assays.

Studies with different acyl-CoA concentrations were carried out to determine the substrate dependencies of FAR1 and FAR2 enzymes. As depicted in Figure 5, TaFAR1 was highly active with 16:0-CoA and showed lower activity with 14:0-CoA or 18:0-CoA. On the other hand, TaFAR2 was distinctly more active with 18:0-CoA than with 14:0-CoA and 16:0-CoA. The different chain length specificities of TaFAR1 and TaFAR2 were in line with results of the *in vivo *expression studies (Figure 2b). They were also supported by competition assays conducted with membrane fractions harboring TaFAR1 or TaFAR2 and a mixture of unlabeled saturated acyl-CoAs with chain lengths of 12 to 20 carbon atoms. TaFAR1 mostly produced 16:0-OH with about 80% of the total alcohol production and lower levels of 14:0-OH and 18:0-OH, whereas TaFAR2 synthesized about 60% of 18:0-OH, 25% of 20:0-OH and smaller amounts of 16:0-OH. Both isozymes were inactive with 12:0-CoA (additional file 6).

**Figure 6:**
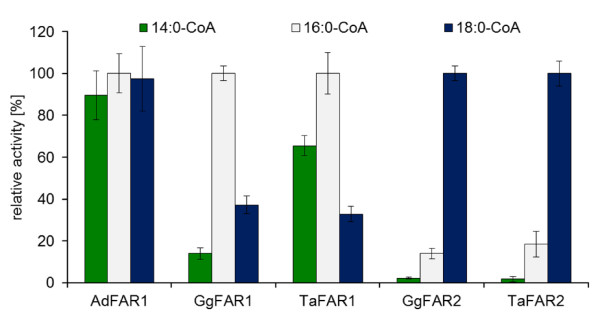
**Substrate specificities of avian FAR enzymes**. *In vitro *assays were conducted with the total membrane fractions of yeast cells harboring one of the avian FAR sequences under optimal conditions and with [1-^14^C]-labeled acyl-CoA thioesters. Results are mean values of assays conducted in duplicate with three independent membrane preparations and represent the sum of synthesized alcohols and aldehydes. 100% correspond to the following FAR activities in nmol*min^-1^*mg^-1 ^membrane protein: AdFAR1: 1.7, GgFAR1: 8.2, TaFAR1: 9.2, GgFAR2: 3.7, TaFAR2: 2.6, control: 0.01.

Figure 6 displays the substrate specificities of FAR1 and FAR2 from barn owl compared to those of the other avian enzymes. The acyl-CoA specificities of FAR from chicken were similar to those of the respective enzymes from barn owl. The FAR1 enzymes were more active with 16:0-CoA than with 14:0-CoA and 18:0-CoA, only AdFAR1 of goose was almost equally active with all substrates. On the other hand, the FAR2 enzymes showed the highest activity with 18:0-CoA. The corresponding studies of FAR1 with 10:0-CoA and 12:0-CoA gave low alcohol formation rates (with TaFAR1 up to 1.68 nmol 10-OH*2 h^-1^*mg^-1 ^protein).

**Figure 7:**
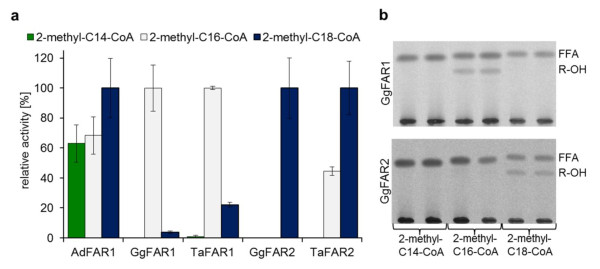
**(a) Activities of avian FAR enzymes with branched-chain acyl-CoA thioesters**. The total membrane fractions of yeast cells harboring one of the avian FAR sequences were used as enzyme source and [1-^14^C]-labeled 2-methyl-branched acyl-CoA thioesters as substrate. Results are mean values of assays conducted in duplicate with three independent membrane preparations and represent the sum of synthesized alcohols and aldehydes. 100% correspond to the following FAR activities in pmol*min^-1^*mg^-1 ^protein: AdFAR1: 25, GgFAR1: 170, TaFAR1: 200, GgFAR2: 60, TaFAR2: 18, control: 10. **(b) TLC analysis of FAR assay products with branched-chain acyl-CoA thioesters**. Reaction products of assays with yeast membranes harboring either GgFAR1 or GgFAR2 are shown.

To investigate the ability of avian FAR enzymes to form branched-chain fatty alcohols that were detected in the uropygial wax esters of barn owl [30], assays with 3,7,11,15-tetramethyl-C16-CoA (phytanoyl-CoA) and 2-methyl-branched acyl-CoAs, with chain lengths of 14 to 18 carbon atoms, were carried out with membrane fractions harboring avian FAR enzymes. As Figure 7 displays, GgFAR1 and TaFAR1 were active with 2-methyl-C16-CoA whereas AdFAR1 as well as the FAR2 enzymes exhibited low activities with 2-methyl-C18-CoA, compared to the empty vector control. In total, these activities were distinctly lower than those with saturated straight-chain acyl-CoAs and aldehyde synthesis could not be observed. No reductase activity was detected with phytanoyl-CoA.

### Expression of GgFAR1 and GgFAR2 in different tissues of chicken

**Figure 8:**
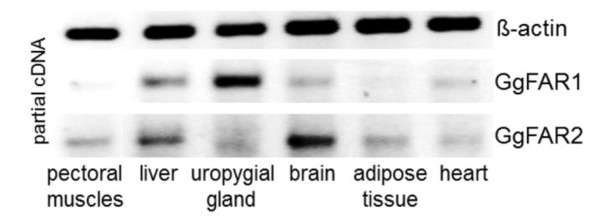
**Semi-quantitative expression analyses of GgFAR1 and GgFAR2 sequences in chicken tissues**. Partial cDNA sequences were amplified by RT-PCR using 1 μg of total RNA isolated from different tissues as template. Synthesis of partial β-actin cDNA was carried out as a control for RNA load. By reactions without reverse transcriptase DNA contamination was excluded.

To investigate whether the expression of avian FAR sequences was restricted to the uropygial gland, the expression patterns of FAR1 and FAR2 were analyzed in the tissues of pectoral muscles, liver, uropygial gland, brain, adipose tissue and heart from domestic chicken by semi-quantitative RT-PCR. As depicted in Figure 8, FAR transcripts were detectable in various tissues, but FAR1 showed an expression pattern clearly different from that of FAR2. Expression of FAR1 was mainly demonstrated in the tissue of uropygial gland, while the highest expression of FAR2 was found in brain tissue.

## Discussion and Conclusion

We report on the functional characterization of fatty acyl-CoA reductases from birds. Based on the sequence similarity between FAR proteins, the respective cDNAs from chicken, barn owl and goose were cloned. Sequence similarities between the avian enzymes in conjunction with their expression pattern and their enzymatic properties suggest that they represent two different groups, termed FAR1 and FAR2. While FAR1 enzymes preferred chain lengths of 15 and 16 carbon atoms, FAR2 enzymes showed a pronounced preference for C18 acyl groups. In that way avian FAR2 enzymes resemble the corresponding mammalian isozymes, while FAR1 enzymes of birds have acyl-CoA specificities different from those of mammalian FAR enzymes [12]. Within the avian FAR1 group only the enzyme of goose was found to have a relaxed chain length specificity. It displayed very similar activities with C14 to C18 acyl groups and thus combined the properties of both FAR1 and FAR2 of chicken and owl. Perhaps geese express a FAR1 gene only in their uropygial glands. That would explain why we succeeded in cloning of FAR1 but not of FAR2 transcripts from this gland tissue. Expression studies with goose tissues will show whether this assumption holds true.

FAR assays with 2-methyl-branched acyl-CoA thioesters gave activity patterns with the different isozymes (Figure 7) similar to those obtained with respective unbranched acyl-CoAs (Figure 6). Again the FAR1 enzymes of both chicken and barn owl were most active with the C16 acyl group while the FAR2 enzymes specifically used the C18 acyl group. Regardless of the bird species, specific activities of the FAR enzymes were about fifty times lower with branched than with unbranched acyl-CoA thioesters. These data suggest that avian FAR can produce branched-chain fatty alcohols only if the enzymes are provided with an acyl-CoA pool largely consisting of branched-chain acyl groups. In view of the very similar properties of FAR enzymes from chicken and barn owl this appears to hold true regardless of whether uropygial glands produce wax ester with branched-chain alcohols, like those of barn owl, or without such alcohols, like chicken and goose [28,30-33]. Uropygial glands have been shown to control their synthesis of multi-branched fatty acids by the substrate pool available to fatty acid synthetase [39]. While malonyl-CoA caused the formation of n-fatty acid, substitution of malonyl-CoA by methylmalonyl-CoA resulted in an efficient synthesis of multi-methyl-branched fatty acid in spite of the fact that the fatty acid synthetase has about 500 times higher activity with malonyl-CoA than with methylmalonyl-CoA *in vitro *[39]. According to these data it is likely that uropygial glands regulate not only fatty acid synthesis but also acyl-CoA reduction by the substrate pool available to the respective enzymes. However, additional regulation mechanisms might be involved as well. Perhaps acyl-CoA synthetases, specifically expressed in the uropygial glands of barn owl and preferentially activating branched-chain fatty acids, can facilitate substrate channeling, but this awaits clarification. Currently we cannot exclude the possibility that birds which secrete wax with branched-chain fatty alcohols possess uropygial gland-specific FAR enzymes unrelated to the known ones but in view of the data so far available this appears unlikely.

Expression studies of FAR sequences in chicken demonstrated that these enzymes are distributed to different tissues. GgFAR1 showed the highest expression level in the uropygial gland whereas GgFAR2 was highly expressed in the brain tissue, suggesting that avian enzymes may be involved in wax ester and ether lipid biosynthesis and perhaps in pheromone production [34]. Enzymes of the FAR family from other taxa can be distributed to numerous tissues as well and are not restricted to one biosynthetic pathway [8,10,12,14].

The characterized enzymes show chain length specificities for middle- to long-chain acyl-CoAs, regardless of whether these substrates are branched or unbranched. Because alcohols with chain lengths between 6 and 22 carbon atoms are manufactured for a multiplicity of applications [36], the characterized avian FAR might be used as enzymatic catalysts for the production of such oleochemicals. Engineering of oil crops expressing FAR in combination with corresponding fatty acid synthesizing and activating enzymes could be a prospective approach to reduce the dependency upon fossil oil to produce fatty alcohols. Expression of enzymes involved in the fatty alcohol biosynthetic pathway in combination with wax ester synthases could be a further possibility to design new oil crops for industry.

## Methods

### Bioinformatical tools

To identify avian FAR sequences, amino acid similarity search was carried out with the "basic local alignment search tool" (BLASTP 2.2.25, NCBI) [40,41] using the following queries: human, HsFAR1 [NCBI: NP_115604]; human, HsFAR2 [NCBI: NP_060569.3]; mouse, MmFAR1 [NCBI: NP_080419.2]; mouse, MmFAR2 [NCBI: NP_848912.1] and jojoba, ScFAR [NCBI: AAD38039.1] (for further accession numbers see additional file 1). Protein property analyses were conducted with "TMHMM" [42-44] and "NCBI Conserved Domain Search" [45-47]. For the illustration of conserved domains and transmembrane regions "PROSITE MyDomains" on the ExPASy server was used. Amino acid sequence alignments were carried out with ClustalX2.1 [48] and GeneDoc [49]. Phylogenetic analysis of FAR amino acid sequences was conducted with MEGA5 [50-53], using the Neighbor-Joining method with 1000 bootstrap replicates. The evolutionary distances were computed using the p-distance method and are in the units of the number of amino acid differences per site.

### mRNA preparation

Tissues of domestic chicken and domestic goose were obtained from Putenfarm Peter Ritte in Wegberg-Rickelrath, tissues of barn owl were obtained from the Institute for Biology II, RWTH Aachen. After the frozen tissue was pulverized with mortar and pestle, 3 volumes of TRIZOL^® ^reagent (Invitrogen, Germany) were added and the suspension was vortexed at room temperature for 15 min. One volume of 1-chlor-3-brompropane (Applichem, Germany) was added and the mixture was incubated at room temperature for 10 min. After centrifugation at 14 000 × g and 4°C for 20 min, the RNA of the aqueous phase was precipitated by adding 1 volume isopropanol and incubating the mixture 15 min at room temperature. The RNA was sedimented by centrifugation at 14 000 × g and 4°C for 20 min. After washing with 70% ethanol, the sedimented RNA was dissolved in 100-300 μL distilled water. Integrity of isolated RNA was analyzed by agarose gel electrophoresis, and the concentration of nucleic acids was measured photometrically. mRNA preparation was carried out using Dynabeads^® ^(Invitrogen, Germany).

### Vector construction and yeast transformation

First strand cDNA synthesis of FAR sequences from mRNA of the uropygial glands was carried out using an AMV reverse transcriptase (Fermentas, Germany) and reverse primer of the respective sequences (Rev-FAR1, 5'-TCAGTATCTCATAGTACTGGAGG-3' or Rev-FAR2, 5'-TCAGTGCCTGAGGGTGCTGG-3'). PCR of FAR1 and FAR2 sequences was carried out with a Pfu-polymerase (Fermentas, Germany) and the primers For-FAR1, 5'-CACCATGGTTTCCATACCTGAATATTATG-3' and Rev-FAR1, or For-FAR2, 5'-CACCATGTCTTCAGTCTCAGCTTATTAC-3' and Rev-FAR2 (Eurofins MWG operon, Germany). Sequences were cloned into the Gateway^® ^entry vector pENTR-SD/D-TOPO (Invitrogen), transformed into *Escherichia coli *TOP10 (Invitrogen). Vectors were re-isolated, sequenced (Fraunhofer IME, Aachen, Germany) and the LR-reaction was carried out with the Gateway^® ^Clonase Mix II™(Invitrogen) and the Gateway^® ^yeast expression vector pYES-DEST52 (Invitrogen) containing a galactose inducible promoter (GAL1). Yeast cells of the strain *Saccharomyces cerevisiae *BY4741 Ɗ*dga1 Ɗlro1 *(*MATa, his3Ɗ1, leu2Ɗ0, met15Ɗ0, ura3Ɗ0, lro1–Ɗ::kanMX4, dga1-Ɗ::natMX4*) [54] were transformed with the expression constructs or the empty vector as a control via electroporation. Transgenic yeast strains were grown in synthetic dropout (SD) medium containing 0.068% (w/v) amino acid supplement mixture (CSM) without uracil and leucine (MP Biomedicals, France), 0.5% (w/v) ammonium sulfate (Roth, Germany), 0.17% (w/v) yeast nitrogen base (MP Biomedicals, France), 0.01% (w/v) leucine (Roth, Germany), 2% (w/v) galactose or 2% (w/v) glucose at 28°C.

### Lipid analysis of transgenic yeast cultures

SD-medium with 2% galactose was inoculated with a yeast preculture harboring one of the FAR constructs or the empty vector as a control and was incubated for 72 h at 28°C without or with supplementation of 250 μM fatty acids. Cells of 10 ml culture were harvested, washed twice with distilled water, dried and after adding of 125 nmol dodecanoic acid and dodecanol (Sigma-Aldrich, Germany) as internal standards the transmethylation of fatty acids was carried out with 2 mL 0.5 M H_2_SO_4 _and 2% 2,2-dimethoxypropane in methanol at 80°C for 1 h. Fatty acid methyl esters (FAME) and fatty alcohols were extracted with 3 mL heptane and analyzed via gas chromatography. Derivatization of fatty alcohols was carried out with 1:1 (v/v) heptane/N, O-bis(trimethylsilyl)trifluoroacetamide (BSTFA) (Roth, Germany) at 70°C for 1 h. Solvents were removed under a stream of nitrogen at room temperature, lipids were dissolved in heptane and analyzed via gas chromatography. Total lipids of transgenic yeast cells were extracted according to Bligh & Dyer [55] and analyzed by TLC (silica gel plate, 60 Å, Merck; Germany) in heptane/diethylether/acetic acid (90:60:1, v/v/v). Lipids were visualized by staining with dichlorofluorescein and identified by comparison with external standards.

### Gas chromatography (GC)

Gas chromatographic analysis was carried out with a HP 6890 gas chromatographic system equipped with the column OPTIMA-5MS (Macherey & Nagel, Germany) and a flame ionization detection (FID) system. The following temperature program was used for FAME and fatty alcohol analysis (2 min at 120°C, then 10°C per min up to 150°C; then 3°C per min up to 270°C; then 10°C per min up to 300°C, hold 1 min at 300°C; with a total column flow of 1.0 mL per min and 1 bar pressure; N_2 _as carrier gas). Identification of the analytes was carried out by comparison of the retention time with those of the external alcohol standards of different chain lengths and degrees of saturation (Sigma-Aldrich, Germany) and in addition by the derivatization of fatty alcohols with BSTFA that resulted in a shift of retention time compared to the free fatty alcohols.

### Preparation of total membrane fractions of transgenic yeast strains

200 mL of induced transgenic yeast cultures were incubated for 16 h at 28°C. Cells were harvested and washed in 20 mL buffer TH (50 mM TRIS/H_2_SO_4_, pH 7.4) and subsequently frozen at -20°C for 10 min. Glass beads (0.75-1 mm diameter) and 2 mL buffer were added and cells were vortexed for 5 min, centrifuged at 1300 × g and 4°C for 5 min. The supernatant was transferred into a new tube and the latter steps were repeated two times. The supernatants were pooled and sonicated twice for 30 s on ice. Cell debris were sedimented at 2500 × g and 4°C for 15 min and the resulting supernatant was centrifuged at 140 000 × g and 4°C for 1 h. Pelletized yeast membranes were resuspended in buffer TH, quick-frozen in liquid nitrogen, and stored at -80°C. Protein concentration was determined [56].

### Synthesis of [1-^14^C]-labeled branched-chain fatty acids

[1-^14^C]2-Methyltetradecanoic, 2-methylhexadecanoic and 2-methyloctadecanoic acids were prepared by α-methylation of the corresponding [1-^14^C]-labeled fatty acids via the sequence carboxylic acid → acyl chloride → diazoketone → chloroketone → 2-methylcarboxylic acid essentially as described [57]. Purification by reversed-phase HPLC (solvent system, acetonitrile/water/acetic acid 85:15:0.01, v/v/v) afforded > 98% pure materials having a specific radioactivity of 0.622 GBq/mmol.

[1-^14^C]3,7,11,15-Tetramethylhexadecanoic acid (phytanic acid) was synthesized starting with 2,6,10,14-tetramethylpentadecanoic acid (pristanic acid; Lipidox Co., Stockholm, Sweden) by the sequence carboxylic acid → primary alcohol → bromide → ^14^C-nitrile → ^14^C-carboxylic acid. The material was purified by reversed-phase HPLC (solvent system, acetontrile/acetic acid 100:0.01, v/v) to afford > 98% pure material having a specific radioactivity of 0.622 GBq/mmol.

### *In vitro *FAR assays

FAR assays were routinely carried out with [1-^14^C]-labeled acyl-CoA thioesters namely 14:0-CoA, 2.04 GBq/mmol; 18:0-CoA, 2.04 GBq/mmol (Biotrend, Germany); 16:0-CoA, 2,22 GBq/mmol (Perkin Elmer, Germany); 2-methyl-branched-CoAs, 0.62 GBq/mmol, 3,7,11,15-tetramethylhexadecanoyl-CoA, 0.17 GBq/mmol, 10:0-CoA, 0.08 GBq/mmol, 12:0-CoA, 0.7 GBq/mmol (acyl-CoA synthesis carried out by Prof. Sten Stymne and his work group). Reaction mixture contained, in a total volume of 50 μl, 2-10 μg membrane protein, 20 μM acyl-CoA, 5 mM NADPH (Sigma-Aldrich, Germany), 1 mM MgCl_2_, 16 μM BSA, 25 mM sodium-phosphate-buffer, pH 6.5 (FAR1) or 25 mM sodium-citrate-buffer, pH 5.5 (FAR2). After 10 min incubation at 37°C reaction products were extracted with 250 μL chloroform/methanol (1:1, v/v) and 100 μL 0.9% (w/v) NaCl solution. The suspension was mixed and, after a brief centrifugation, 80 μL of the chloroform phase were analyzed by TLC. [^14^C]-labeled reaction products were visualized by the phosporimager system FLA3000 (Fujifilm), identified by external standards and quantified with the multi-purpose scintillation counter LS 6500 (Beckman Coulter).

FAR assays with non-labeled substrates were carried out in the similar way but 20 μM [1-^14^C]-labeled acyl-CoA was substituted by 20 μM of 12:0-CoA, 14:0-CoA, 16:0-CoA, 18:0-CoA, (Sigma-Aldrich, Germany) and 20:0-CoA (Avanti, USA) and incubation time was extended to 4 h and the assay volume was increased (500 μL). Extracted reaction products were transmethylated and analyzed by GC.

### Semi-quantitative expression analysis of GgFAR1 and GgFAR2 in chicken

1 μg of total RNA isolated from tissues of pectoral muscles, liver, uropygial gland, brain, heart and adipose tissue of chicken were digested with DNase I (Fermentas, Germany) and reverse transcription was carried out with AMV reverse transcriptase (Fermentas) using an oligo-(dT)-primer. PCR was conducted with 1 μL first strand cDNA as template, Taq-polymerase (Genecraft, Germany) and primers for GgFAR1 (Forward: 5'-GACACCAGAAGCACGGATAG-3', Reverse: 5'-TCCAGTTCAGGCTGTGTAAG-3', 126 bp fragment), GgFAR2 (Forward: 5'-CTCCTGCCATACTCTATGAC-3', Reverse: 5'-GACTGGGTGGAGAAATACTG-3', 118 bp fragment) and β-actin (Forward: 5'-ACCTGAGCGCAAGTACTCTG-3', Reverse: 5'-ACAATGGAGGGTCCGGA-3', 114 bp fragment). Reactions without reverse transcriptase were carried out to exclude DNA contamination. Amplified fragments were analyzed by agarose gel electrophoresis.

## Abbreviations

12:0-OH: 1-dodecanol; 13:0-OH: 1-tridecanol; 14:0-OH: 1-tetradecanol; 15:0-OH: 1-pentadecanol; 16:0-OH: 1-hexadecanol; 17:0-OH: 1-heptadecanol; 18:0-OH: 1-octadecanol; 19:0-OH: 1-nonadecanol; 20:0-OH: 1-eicosanol; 16:1-OH: 1-hexadecenol; 18:1-OH: 1-octadecenol; 10:0-CoA: decanoyl-CoA; 12:0-CoA: dodecanoyl-CoA; 14:0-CoA: tetradecanoyl-CoA; 16:0-CoA: hexadecanoyl-CoA; 18:0-CoA: octadecanoyl-CoA; 20:0-CoA: eicosanoyl-CoA; 2-methyl-C14-CoA: 2-methyltetradecanoyl-CoA; 2-methyl-C16-CoA: 2-methylhexadecanoyl-CoA; 2-methyl-C18-CoA: 2-methyloctadecanoyl-CoA; 13:0: tridecanoic acid; 15:0: pentadecanoic acid; 17:0: heptadecanoic acid; 19:0: nonadecanoic acid.

## Authors' contributions

JH designed, conducted and analyzed the experiments and prepared the manuscript; EMB contributed to the design and the conduction of experiments and revised the manuscript; MF coordinated and supervised the study and was involved in preparing the manuscript; JG contributed to RNA preparation and cDNA synthesis and gave technical support and helpful advices, MH carried out synthesis of branched-chain fatty acids and wrote the corresponding method section. All authors read and accepted the final manuscript.

## Supplementary Material

Additional file 1 **NCBI accession numbers of FAR proteins**. Click here for file

Additional file 2 **Alignment of FAR1 [NCBI: NP_001026350.1] and GgFAR1 of chicken**. Alignment was carried out with ClustalX2.1 and GeneDoc and displays the substitution of the sequence from amino acid position 319 to position 376 in GgFAR1 compared to FAR1. Click here for file

Additional file 3 **Illustration of the conserved domains and the predicted transmembrane regions**. TaFAR1 and TaFAR2 sequences of barn owl are exemplary shown for avian sequences in comparison to MmFAR1 [NCBI: NP_080419.2] of mouse and AtCER4 [NCBI: NP_567936.5] of Arabidopsis. TM: transmembrane region. Click here for file

Additional file 4 **TLC analyses of the reaction products of FAR1 and FAR2 assays with different acyl-CoA thioesters**. Assays were conducted with the total membrane fractions of transgenic yeast cells expressing one of the FAR sequences from barn owl and chicken and 20 μM labeled 14:0-CoA, 16:0-CoA or 18:0-CoA under standard conditions (ALD: fatty aldehyde, FFA: free fatty acid, R-OH: fatty alcohol). Click here for file

Additional file 5 **Optimization of assay conditions**. **(a) pH-value dependency of TaFAR1 and TaFAR2 activity**. Assays were conducted with the total membrane fractions of transgenic yeast cells and with labeled 16:0-CoA (TaFAR1) or labeled 18:0-CoA (TaFAR2) but buffer and pH were varied namely pH 4.5 to pH 6.0: sodium-citrate-buffer, pH 6.5 to pH 8.0: sodium-phosphate-buffer. Aldehydes and alcohols are given as mean values of two independent assays. **(b) 16:0-CoA dependency of TaFAR1 activity with and without bovine serum albumin**. Assays were run with the given concentrations of labeled 16:0-CoA without and with 16 μM BSA. **(c) Protein linearity**. Assays were carried out with the given mass of membrane protein harboring either TaFAR1 or TaFAR2. Data are the sum of synthesized aldehydes and alcohols and are given as mean values of two independent assays. Click here for file

Additional file 6 **GC analyses of reaction products of FAR competition assays**. Standard assays were run with the total membrane fractions of yeast cells expressing TaFAR1 or TaFAR2 but 20 μM labeled acyl-CoA was substituted by a mixture of unlabeled acyl-CoA thioesters (12:0-CoA, 14:0-CoA, 16:0-CoA, 18:0-CoA and 20:0-CoA). The volume was increased tenfold and incubation time was extended to 4 h. Extracted lipophilic reaction products were transmethylated and analyzed by GC. (a: 14:0-OH, b: 16:0-OH, c: 18:0-OH, d: 20:0-OH). Click here for file
