# Supplementary material for: Fatty acyl-CoA reductases of birds
Source: BMC Biochem. 2011 Dec 12;12:64. doi: 10.1186/1471-2091-12-64 (PMC3265415; doi:10.1186/1471-2091-12-64)
Supplement: Additional file 1 — NCBI accession numbers of FAR proteins. [file 1471-2091-12-64-S1.PDF]

| Used Abbreviation | NCBI accession numbers |
|---|---|
| AdFAR1 | JN638548 |
| AmFAR1 | NP_001180219.1 |
| AtCER4 | NP_567936.5 |
| AtFAR1 | NP_197642.1 |
| AtFAR4 | NP_190040.3 |
| AtFAR5 | NP_190041.2 |
| AtFAR6 | AEE79553.1 |
| AtFAR8 | NP_190042.2 |
| AtMS2 | AEE75132.1 |
| BmFAR | NP_001036967.1 |
| EgFAR | ADI60057.1 |
| FAR1 | NP_001026350.1 |
| FAR2 | XP_417235.2 |
| HsFAR1 | NP_115604.1 |
| HsFAR2 | NP_060569.3 |
| MmFAR1 | NP_080419.2 |
| MmFAR2 | NP_848912.1 |
| OnFARa | ACY07547.1 |
| OnFARb | ACY07546.1 |
| OsFARVIII | ACJ06520.1 |
| ScFAR | AAD38039.1 |
| TaFAR1 | JN638549 |
| TaFAR2 | JN638550 |
| TaTAA1a | CAD30693.1 |
| YeFARI | ADD62438.1 |
| YeFARII | ADD62439.1 |
| YeFARIII | ADD62440.1 |
| YpFARII | ADD62442.1 |
| YrFARII | ADD62441.1 |
