# Supplementary material for: Fatty acyl-CoA reductases of birds
Source: BMC Biochem. 2011 Dec 12;12:64. doi: 10.1186/1471-2091-12-64 (PMC3265415; doi:10.1186/1471-2091-12-64)
Supplement: Additional file 2 — Alignment of FAR1 [NCBI: NP_001026350.1] and GgFAR1 of chicken. Alignment was carried out with ClustalX2.1 and GeneDoc and displays the substitution of the sequence from amino acid position 319 to position 376 in GgFAR1 compared to FAR1. [file 1471-2091-12-64-S2.PDF]

```
                  *        20         *        40         *        60         *
FAR1   : MVSIPEYYEGKNVLLTGATGFMGKVLLEKLLRSCPKVKAVYVLVRPKAGQTPEARIEEITSCKLFDRLREE :  71
GgFAR1 : MVSIPEYYEGKNVLLTGATGFMGKVLLEKLLRSCPKVKAVYVLVRPKAGQTPEARIEEITSCKLFDRLREE :  71

                80         *       100         *       120         *       140
FAR1   : QPDFKEKIIVITSELTQPELDLSNPVKEKLIECINIIFHCAATVRFNETLRDAVQLNVLSTKQLLSLAQQM : 142
GgFAR1 : QPDFKEKIIVITSELTQPELDLSNPVKEKLIECINIIFHCAATVRFNETLRDAVQLNVLSTKQLLSLAQQM : 142

                *       160         *       180         *       200         *
FAR1   : TNLEVFMHVSTAYAYCNRKHIEEVVYPPPVDPKKLMDSLEWMDDSLVNDITPKLIGDRPNTYTYTKALAEY : 213
GgFAR1 : TNLEVFMHVSTAYAYCNRKHIEEVVYPPPVDPKKLMDSLEWMDDSLVNDITPKLIGDRPNTYTYTKALAEY : 213

             220         *       240         *       260         *       280
FAR1   : VVQQEGARLNTAIIRPSIVGASWKEPFPGWIDNFNGPSGLFIAAGKGILRTMRASNGAVADLVPVDVVVNM : 284
GgFAR1 : VVQQEGARLNTAIIRPSIVGASWKEPFPGWIDNFNGPSGLFIAAGKGILRTMRASNGAVADLVPVDVVVNM : 284

              *       300         *       320         *       340         *
FAR1   : TLAAAWYSGVNRPRNIMVYNCTTGGTNPFHWSEVEYHVISTFKRNPLEQAFRRPNVNLTSNHLLYHYWIAV : 355
GgFAR1 : TLAAAWYSGVNRPRNIMVYNCTTGGTNPFHWSEVAYHVYLNYKTNPLDGAIRHPSIVLCSNPLLHQYWTTV : 355

           360         *       380         *       400         *       420
FAR1   : SHKAPAFLYDIYLRITGRSPRMMKTITRLHKAMVFLEYFTSNSWIWNTENMTMLMNQLSPEDKKTFNFDVR : 426
GgFAR1 : RHILPAFSYDVLLKLTGHKPWMMKTITRLHKAMVFLEYFTSNSWIWNTENMTMLMNQLSPEDKKTFNFDVR : 426

            *       440         *       460         *       480         *
FAR1   : QLHWAEYMENYCMGTKKYVLNEEMSGLPAARKHLNKLRNIRYGFNTILVILIWRIFIARSQMARNIWYFVV : 497
GgFAR1 : QLHWAEYMENYCMGTKKYVLNEEMSGLPAARKHLNKLRNIRYGFNTILVILIWRIFIARSQMARNIWYFVV : 497

         500         *
FAR1   : SLCYKFLSYFRASSTMRY : 515
GgFAR1 : SLCYKFLSYFRASSTMRY : 515
```
