# Supplementary figures and images for: Fatty acyl-CoA reductases of birds
Source: BMC Biochem. 2011 Dec 12;12:64. doi: 10.1186/1471-2091-12-64 (PMC3265415; doi:10.1186/1471-2091-12-64)

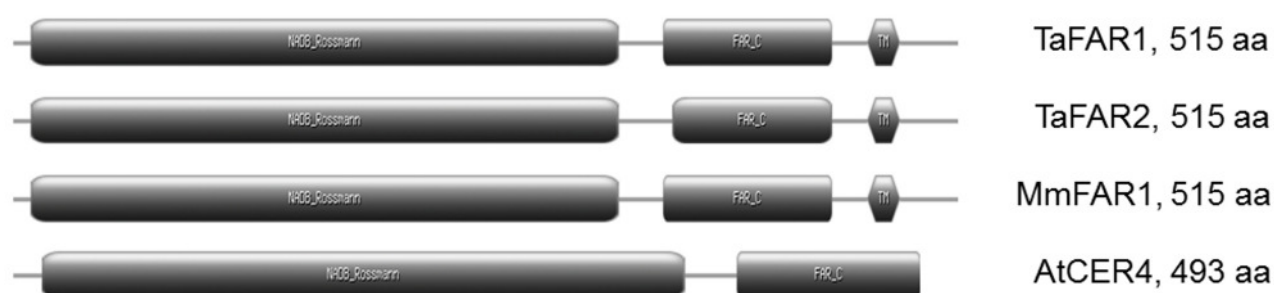

Supplement: Additional file 3 — Illustration of the conserved domains and the predicted transmembrane regions. TaFAR1 and TaFAR2 sequences of barn owl are exemplary shown for avian sequences in comparison to MmFAR1 [NCBI: NP_080419.2] of mouse and AtCER4 [NCBI: NP_567936.5] of Arabidopsis. TM: transmembrane region. [file 1471-2091-12-64-S3.PDF]

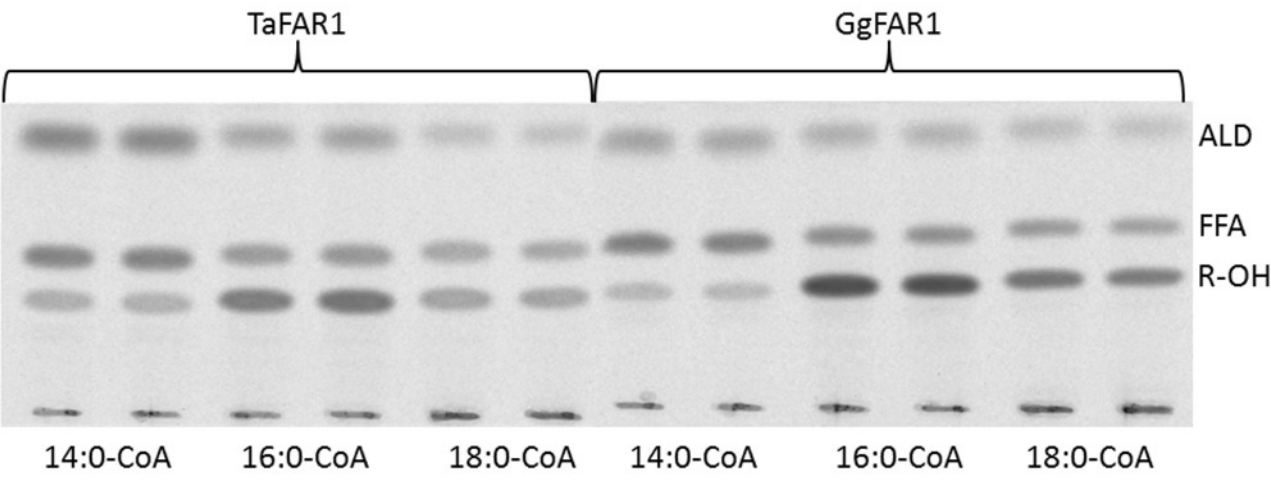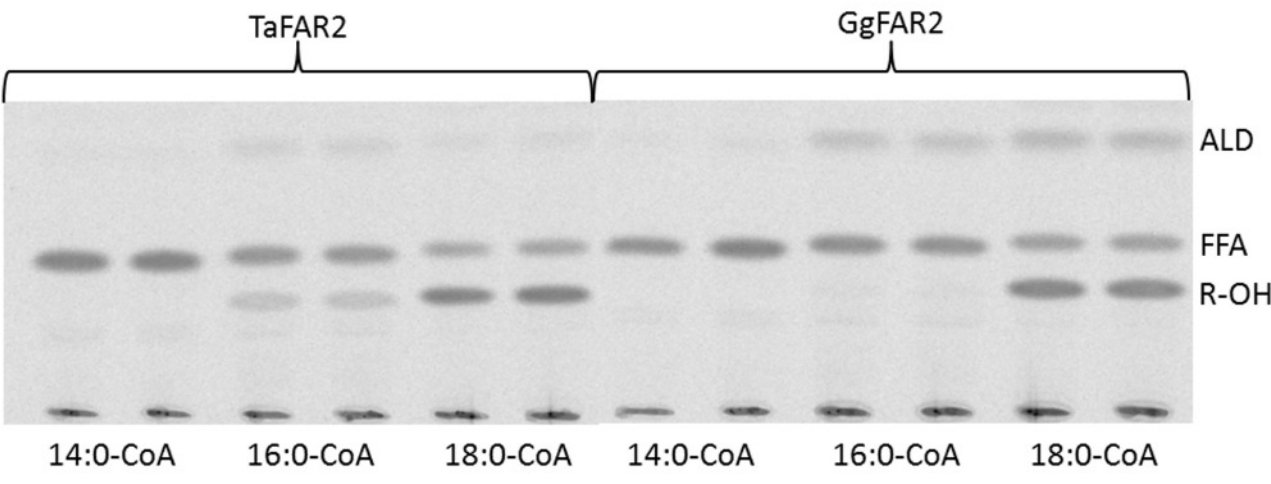

Supplement: Additional file 4 — TLC analyses of the reaction products of FAR1 and FAR2 assays with different acyl-CoA thioesters. Assays were conducted with the total membrane fractions of transgenic yeast cells expressing one of the FAR sequences from barn owl and chicken and 20 μM labeled 14:0-CoA, 16:0-CoA or 18:0-CoA under standard conditions (ALD: fatty aldehyde, FFA: free fatty acid, R-OH: fatty alcohol). [file 1471-2091-12-64-S4.PDF]

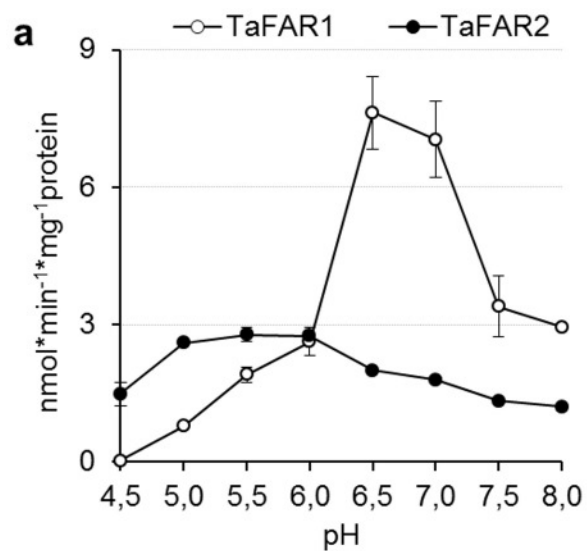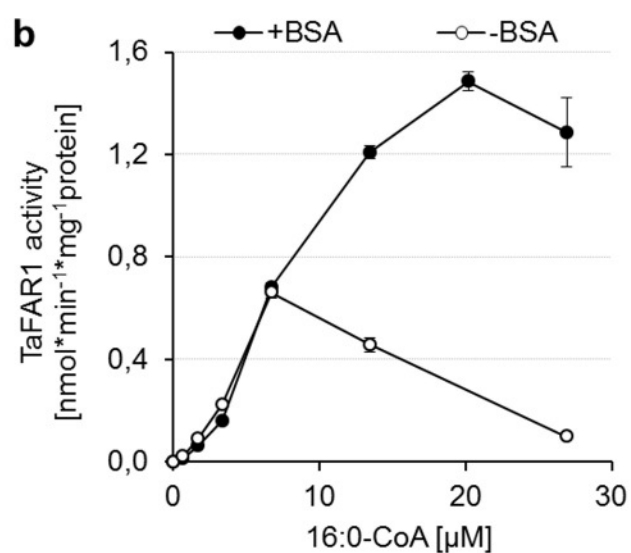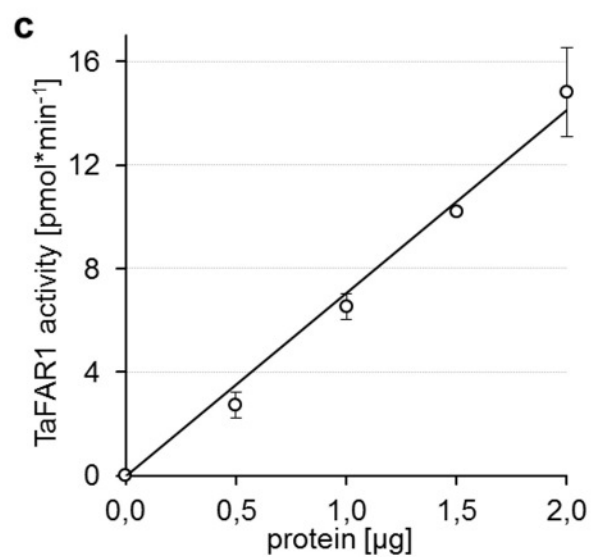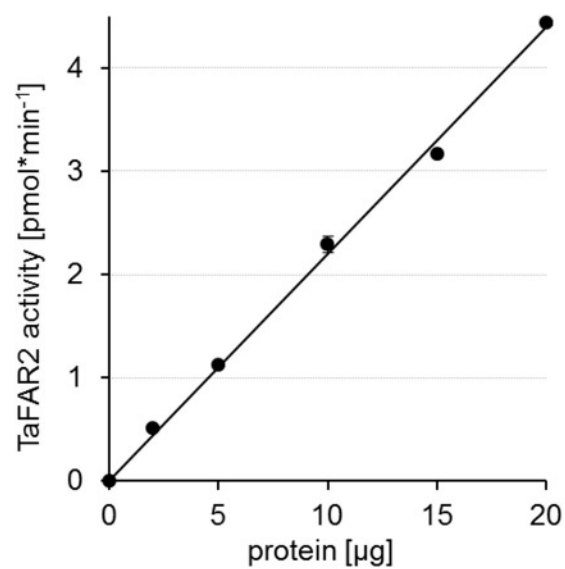

Supplement: Additional file 5 — Optimization of assay conditions. (a) pH-value dependency of TaFAR1 and TaFAR2 activity. Assays were conducted with the total membrane fractions of transgenic yeast cells and with labeled 16:0-CoA (TaFAR1) or labeled 18:0-CoA (TaFAR2) but buffer and pH were varied namely pH 4.5 to pH 6.0: sodium-citrate-buffer, pH 6.5 to pH 8.0: sodium-phosphate-buffer. Aldehydes and alcohols are given as mean values of two independent assays. (b) 16:0-CoA dependency of TaFAR1 activity with and without bovine serum albumin. Assays were run with the given concentrations of labeled 16:0-CoA without and with 16 μM BSA. (c) Protein linearity. Assays were carried out with the given mass of membrane protein harboring either TaFAR1 or TaFAR2. Data are the sum of synthesized aldehydes and alcohols and are given as mean values of two independent assays. [file 1471-2091-12-64-S5.PDF]

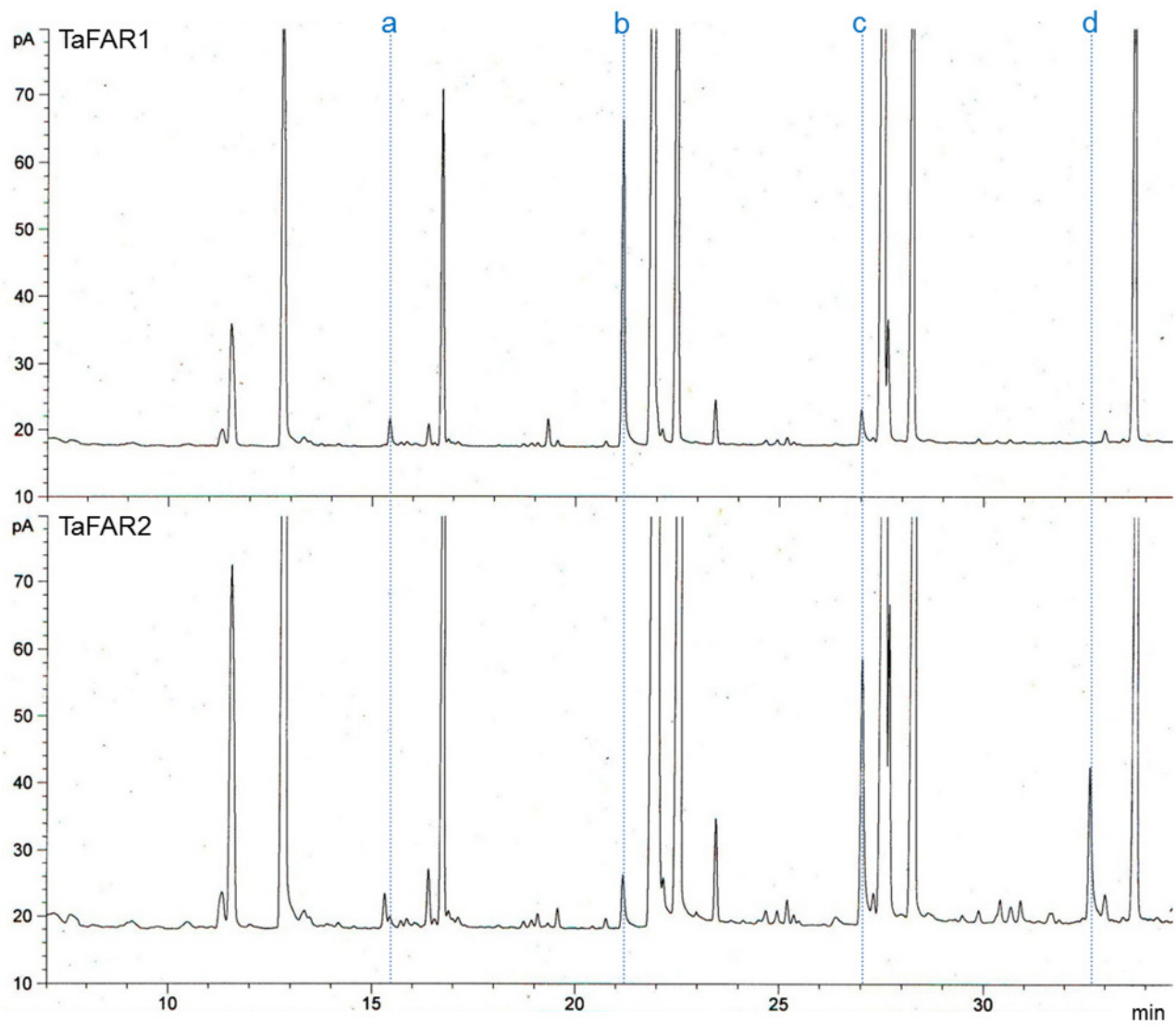

Supplement: Additional file 6 — GC analyses of reaction products of FAR competition assays. Standard assays were run with the total membrane fractions of yeast cells expressing TaFAR1 or TaFAR2 but 20 μM labeled acyl-CoA was substituted by a mixture of unlabeled acyl-CoA thioesters (12:0-CoA, 14:0-CoA, 16:0-CoA, 18:0-CoA and 20:0-CoA). The volume was increased tenfold and incubation time was extended to 4 h. Extracted lipophilic reaction products were transmethylated and analyzed by GC. (a: 14:0-OH, b: 16:0-OH, c: 18:0-OH, d: 20:0-OH). [file 1471-2091-12-64-S6.PDF]
